# Gold Mono- and Bis-N-heterocyclic Carbenes Based on mRNA cap0

**DOI:** 10.1021/acsomega.5c06425

**Published:** 2025-10-02

**Authors:** Giulia Francescato, Giulia Orsini, Ana Petronilho

**Affiliations:** Instituto de Tecnologia Química e Biológica António Xavier, Universidade Nova de Lisboa. Av. da República, 2780-157 Oeiras, Portugal

## Abstract

Eukaryotic mRNA contains a cap structure that consists of a methylated guanosine (mRNA cap0) connected to the first transcribed nucleotide by an unusual 5′-5′-triphosphate bridge. Herein, we describe the synthesis of gold mono- and bis-N-heterocyclic carbenes derived from 7-methylguanosine at room temperature. The compounds can be synthesized directly from 7-methylguanosine, and the synthesis does not require the protection of the hydroxyl groups of the ribose to be effective. The synthesis was also performed successfully with the acetate protected nucleoside, but deprotection of the sugar was not effective. The compounds are stable under air, both in the solid state and in solution. Gold monocarbene **1** retains its ability to form Watson–Crick base pairs with cytosine, without interference of the acetate protecting groups.

## Graphical abstract

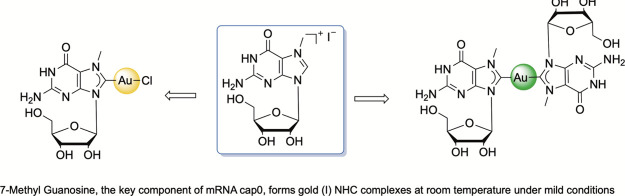

## Introduction

One of the most important characteristics of eukaryotic mRNA is the presence of a cap structure.^1,2^ This cap consists of an N7-methylated guanosine connected to the first transcribed nucleotide by an unusual 5′-5′-triphosphate bridge.^3^ The cap is recognized by specific proteins, such as eukaryotic initiation factor 4E (eIF4E), involved in the initiation of translation.^4,5^ Understanding all of the aspects of the reactivity of this cap is pivotal, not only to determine possible mRNA behavior but also to allow for suitable modifications for the development of cap analogues.^6–8^ Modified cap analogues can have important potential uses such as inhibitors of cap-dependent translation,^9^ inhibitors of decapping enzymes, or fluorescent probes to evaluate interactions with cap-specific proteins.^10–12^ Synthetic cap analogues can also be employed to modify the 5′-end of mRNA for developing therapeutic mRNAs.^13,14^ Thus, 7-methylguanosine is a promising site for introducing modifications into the cap structure. Yet, the development of synthetic methods for cap structure modification is challenging due to the difficulty of finding selective methods that are site specific. In this context, 7-methylguanosine bears a very acidic C8–H, due to the formation of an ylide upon proton loss,^15,16^ with a reactivity characteristic of imidazolium salts and N-heterocyclic carbene precursors.^17,18^ Like imidazolium salts, 7-methylguanosine catalyzes benzoin condensation^19^ and single-stranded DNA labeled with N7-methylguanine reacts with acetone,^16^ presumably through the formation of an N-heterocyclic carbene intermediate. Thus, exploring the ability of 7-methylguanosine to form N-heterocyclic carbene (NHC) ligands seems to be a good strategy to induce modifications at mRNA caps.

Organometallic derivatization of nucleosides^20–24^ has emerged as a promising strategy to enhance their therapeutic profiles. Our group has been actively investigating the derivatization of nucleobases and nucleosides with metal complexes, aiming to develop effective methods for the formation of organometallic nucleosides and further understand their properties once metalated. In our pursuit to examine the potential of nucleobases and nucleosides to form N-heterocyclic carbenes,^25–30^ we aimed at exploring the reactivity of 7-methylguanosine with transition metals.

We have recently reported the synthesis of platinum NHCs based on 7-methylguanosine and derivatives by unassisted C–H activation.^26^ In these reactions, we made use of the acidic C8–H bond to promote oxidative addition to Pt(0). Importantly, we observed that metalation increases considerably the stability of 7-methylguanosine toward hydrolysis. The reactivity found for 7-methylguanosine contrasts with that of imidazolium salts,^31,32^ for which oxidative addition is limited, due to competitive reductive elimination. The reaction, however, requires higher temperatures for it to be effective. Herein, we describe the reactivity of 7-methylguanosine with gold­(I) at room temperature to form mono- and bis-NHC gold complexes, by base-assisted deprotonation.

## Results and Discussion

Earlier examples from our group showed that the metalation of unprotected purine nucleosides leads to a myriad of compounds,^25–27^ and the protection of the ribose generally leads to more^25^ selective reactions. Guanosine was protected with acetate, and protection was followed by methylation at N7 with methyl iodide in DMA, yielding **7-MeG**_**OAc**_. The ligand precursor **7-MeG**_**OAc**_ was reacted with Au­(SMe_2_)Cl at room temperature, in DMF (or alternatively DMSO) in the presence of K_2_CO_3_ (Scheme 1), following procedures typically employed for the synthesis of gold NHC complexes.^33–35^ ^1^H NMR analysis of the reaction after this time showed the complete consumption of **7-MeG**_**OAc**_ as evidenced by the disappearance of the C–H(8) signal. It was possible to identify two compounds (**1** and **2)**, due to the presence of two N7-methyl groups at 3.98 and 4.09 ppm in a 9:1 ratio, respectively, and also two sets of signals associated with the ribose protons.

Given the well-established propensity of gold compounds to form both mono- and bis-NHCs,^25^ we hypothesized that the minor compound was a gold bis-NHC complex and the major compound was a monocarbene.

**Scheme 1:**
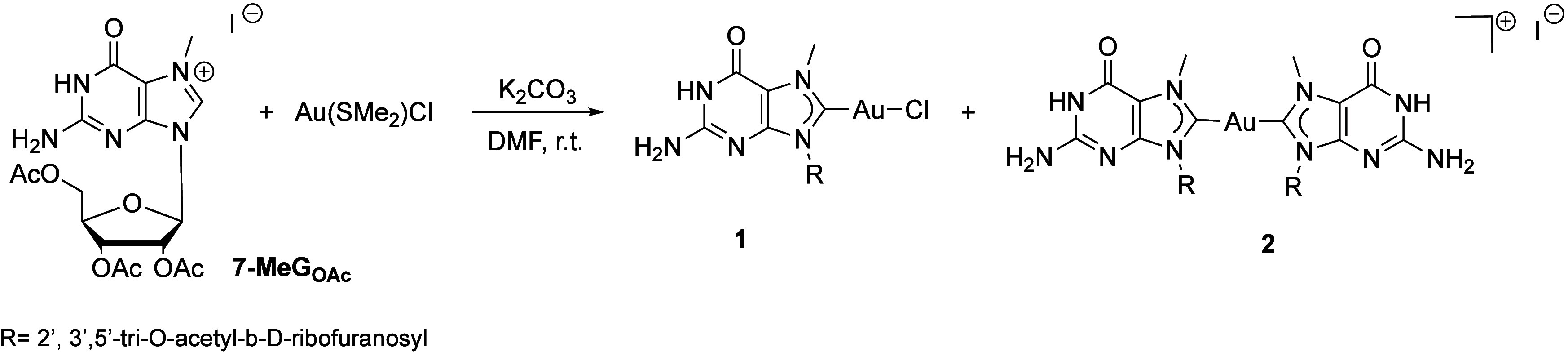
Synthesis of Gold­(I) Complexes **1** and **2**

In the ^13^C NMR, the C8 values for compounds **1** and **2** were difficult to detect, but 2D HMBC clearly indicated cross-peaks with the N7-methyl group of **1,** at 172.2 ppm, while for compound **2,** the cross-peak was observed at 184.7 ppm, in good agreement with values typically found for gold mono- and bis-NHCs.^34–39^ Conducting a diffusion-ordered spectroscopy revealed that the minor compound had a higher molecular weight, which agrees well with the formation of mono- and biscarbene complexes of gold­(I). Evaluation of the mixture by mass spectrometry was nonetheless inconclusive.

We then focused on the separation of the two compounds obtained. Due to the low solubility of the mixture, chromatographic separation was revealed to be not feasible. Attempts to precipitate or induce crystallization of the major compound were also unsuccessful. We then performed the reaction in the presence of a source of chloride. Thus, the reaction was conducted with 10 equiv of NaCl, but also in this case, a mixture was obtained. The reaction was also performed using a ligand precursor bearing BF_4_ instead of iodide. However, when reacted with gold­(I) under the same conditions, a mixture similar to that obtained with the iodide salt **7-MeG**_**OAc**_ was formed.

We performed several modifications of the reaction conditions, aiming to make the reaction more selective. For example, changing the solvent to acetonitrile, toluene, or acetone proved ineffective. For the reactions with acetone, we employed the conditions described by Nolan and Collado,^40,41^ with which we expected the generation of monocarbene **1** through the prior formation of an aurate anion, formed after the loss of the dimethylsulfide ligand from the gold­(I) precursor.^40^ However, for **7MeG**_**OAc**_, the reactions were not successful; we did not observe the formation of an aurate and obtained once again a mixture of mono- and biscarbene complexes. Attempts to form the gold NHCs employing transmetallation from the corresponding silver compounds were also pursued. **7MeG**_**OA**_ was reacted with silver acetate in methanol, but the corresponding NHC was not obtained. A highly insoluble precipitate is formed, probably the silver halide salt, since the C8–H bond of the ligand precursor **7MeG**_**OA**_ remains intact in solution. Similar results were obtained with silver oxide. Finally, attempts to oxidize the mixture with both phenyliodide and I_2_, to obtain the corresponding gold­(III) complexes, were also unsuccessful.^42^

Following on this, we opted to determine if it was possible to form selectively compounds **1** and **2** employing variations of the reaction conditions used initially. The reaction was monitored by ^1^H NMR in DMF-*d*_7_. After 10 h, the ^1^H NMR spectrum shows the ligand precursor **7MeG**_**OA**_ and compound **1**, in a 1:1 ratio. As the reaction progresses, compound **1** becomes the major compound, but compound **2** starts to form and can be identified by the appearance of a set of small signals corresponding to the ribose ring at 6.37, 5.99, and 5.70 ppm, respectively. After total consumption of the ligand precursor **7MeG**_**OA**_, the final mixture shows compounds **1** and **2** in a 9:1 ratio. It is thus clear that the formation of complex **2** cannot be avoided while ensuring the complete consumption of **7MeG**_**OA**_. Considering these results, we pursued a different synthetic approach, assuming that the stoichiometry of the reaction could improve selectivity. While this is evident for the formation of bis-NHC complex **2**, for mono-NHC complex **1,** this is not the case. Thus, **7MeG**_**OA**_ was reacted with 2 equiv of Au­(SMe_2_)Cl in the presence of K_2_CO_3_ for 48 h (Scheme 2). During this time, the formation of a pink-brownish precipitate was observed. After 48 h, the precipitate was isolated. ^1^H NMR analysis of the solids did not show any identifiable compound.

**Scheme 2:**
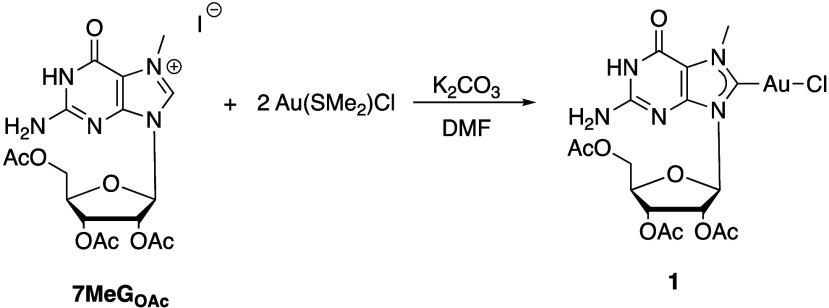
Synthesis of Gold­(I) NHC Complex **1**

The mother liquor was then precipitated with Et_2_O, and ^1^H NMR analysis of the white solid that formed confirms the formation of compound **1**. ^1^H NMR shows the methyl group of N7 at 3.98 ppm, as described earlier. The exclusive formation of complex **1** also made it possible to identify all the ribose ring protons: the anomeric H1′ appears as a doublet at 6.27 ppm. This proton couples with H2′, presenting with ^3^J_H‑1′,H‑2′_ = 5.12 Hz. In the ^13^C­{^1^H}-NMR spectra, the carbenic C8 resonates at 172.2 ppm, conforming with the value described for the mixture of **1** and **2**. The ribose ring carbons resonate at 89.8 for the C1′ and C4′ at 79.6 ppm and 71.3, 69.9, and 62.7 ppm for C2′, C3′, and C5′, respectively. Compound **1** is partially soluble in DMSO and DMF, and it can be fully solubilized upon heating.

To obtain compound **2**, 2 equiv of **7MeG**_**OA**_ and K_2_CO_3_ were used instead, while the remaining reaction conditions were maintained.

As the reaction progressed, a flocculent white precipitate formed. After 48 h, **7MeG**_**OA**_ was totally consumed. The white precipitate was isolated, and the mother liquor was layered with Et_2_O, leading to the formation of a precipitate. Analysis of both solids by NMR indicates that compound **2** is the only compound present in both cases, confirming the success of the reaction. ^1^H NMR analysis shows the methyl group of N7 at 4.09 ppm, as described previously. As was the case with compound **1**, the formation of the biscarbene as the only product of the reaction allowed for the identification of the ribose ring protons. Thus, H1′ resonates at 6.37 ppm, H2′ at 5.99, and H3′ at 5.70 ppm. H4′ and one of the two diasterotopic H5′ appear overlapped between 4.48 and 4.32 ppm, while the remaining H5′ resonates at 4.23 ppm. The ^13^C­{^1^H} NMR spectrum clearly shows the signal at 184.8 ppm, corresponding to the C8 carbenic carbon. This value is consistent with that described previously for the mixture of compounds and also agrees well with those reported for other gold­(I) biscarbenes.^34,36^ The ribose carbons follow the same order found for **1**, with C1′ at 90.1 ppm, followed by C4′ at 79.6 ppm, and then C2′, C3′, and C5′ at 72.4, 69.9, and 63.2 ppm, respectively. Regarding the relative position of the NHC ligands, nuclear Overhauser enhancement spectroscopy analysis shows a cross-peak between the CH_3_ group in N7 and the H1′ position of the ribose supporting the ligand orientation depicted in Scheme 3. Compound **2** is highly insoluble in several solvents; for example, in DMSO, compound **2** only dissolves partially and full solubilization can only be achieved upon heating.

**Scheme 3:**
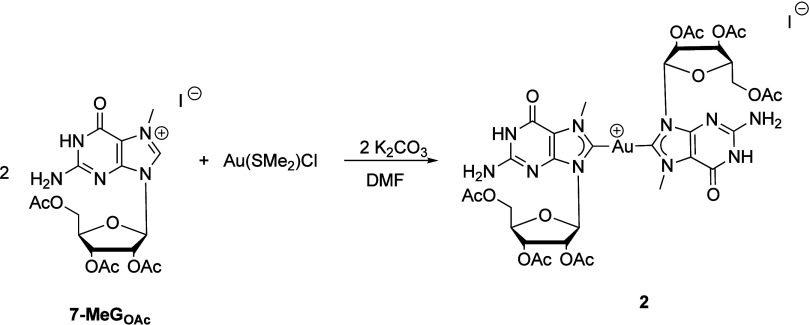
Synthesis of Gold­(I) Bis-NHC Complex **2**

Attempts to deprotect compounds **1** and **2** were not successful. Compound **1** was stirred in the presence of HCl, but deprotection was not successful. As for compound **2**, due to the poor solubility, deprotection was also revealed to be unattainable. Previous work from our group on metal complexes bearing nucleosides has shown that guanosine and adenosine proligands require protection when reacting by C–X or C–H oxidative addition to Pt or Pd (0).^25–27,29^ Contrastingly, for similar reactions, uridine proligands react cleanly without requiring the protection of the sugar.^28^

Given the difficulties in finding a suitable deprotection method, most probably driven by the low solubility of the compounds in suitable solvents for deprotection, we performed the reaction with the unprotected guanosine ligand precursor **7MeG.** Following a similar strategy as that used previously to achieve selectivity, 2 equiv of proligand **7MeG** was used for the synthesis of the biscarbene and 2 equiv of Au­(SMe_2_)Cl to obtain the deprotected monocarbene. The reactions proceeded successfully, and it was possible to obtain compounds **3** and **4** selectively (Figure 1). This contrasts with what was observed previously for guanosine-based ligands in oxidative addition processes.

**Figure 1:**
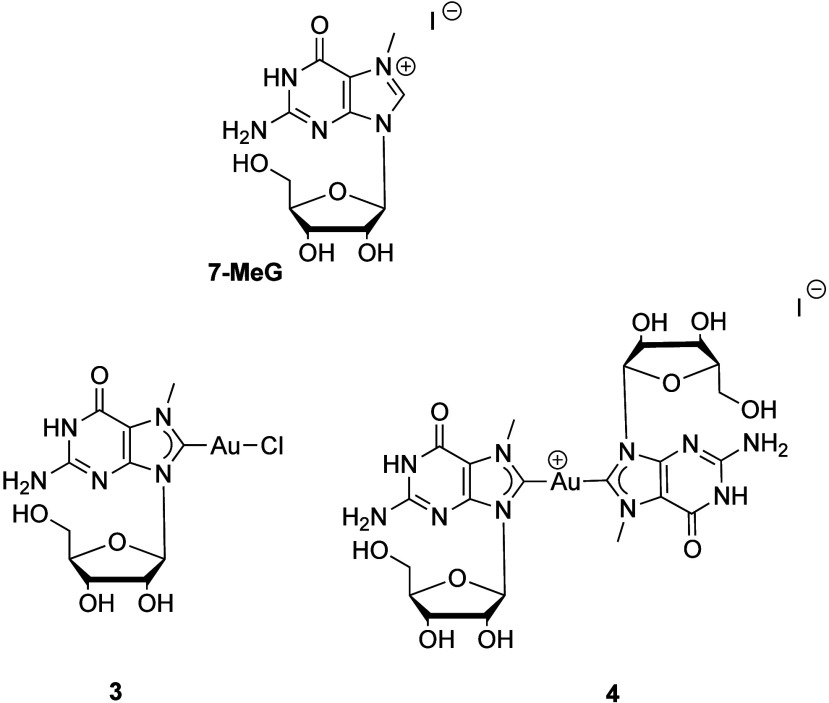
Gold­(I) mono- and bis-NHC complexes **4** and **5**.

Compound **3** shows a similar NMR to that of **1**, with the N7-Me group resonating at 3.99 ppm in ^1^H NMR, while the carbenic carbon can be found at 172.4 ppm in ^13^C­{^1^H} NMR. Compound **4** shows the N7-Me at 4.11 ppm in ^1^H and C8 at 185.1 ppm in ^13^C. Both compounds are poorly soluble in most solvents, as was the case with the corresponding protected compounds. Indeed, to be able to fully characterize compound **4**, the compound was suspended in DMSO and heated, and ^13^C was performed at 50 °C to avoid precipitation during acquisition.

Compounds **1–4** are stable for weeks both in the solid state and in solution. Indeed, solutions of **1** in DMSO-*d6* do not show signs of decomposition even after 2 weeks. Also, heating the sample at 60 °C for 3 days does not lead to any noticeable degradation. Stability under mildly acidic conditions was also examined. To solutions of **1** in DMSO-*d*_6_ was added 100 μL of a HCl solution (pH 2), and the evolution of the solution was monitored through ^1^H NMR. The compound remains stable with no signs of decomposition for at least 48 h. A similar experiment employing a pH 1 HCl solution was also performed. In this case, we observe immediately the formation of a small amount of the bis-NHC **2**, which does not increase over time (after 3 and 5 h). After 24 h, the NMR spectra do not show fully the mono or the bis-NHC, but we also do not observe any compounds resulting from degradation. Importantly, free 7-methylguanosine, as the hypothetical result of protonation of the NHC, was not detected, nor was free ribose resulting from hydrolysis (see the SI).

The reactivity of 7,9-dimethylguanine (**7,9-DMG**) with gold was also explored. When the reaction is performed with 7,9-dimethylguanine iodide salt instead of **7MeG** (where formally the ribose is replaced by a methyl group at N9), no gold NHC is formed and the proligand is recovered. By contrast, as previously reported, when 7,9-dimethylguanine reacts with Pt­(PPh_3_)_4_ by C–H oxidative addition, the corresponding NHC is formed cleanly and in good yields, as we reported previously.^26^ We also examined the reactivity of the corresponding nucleotide, 7-methylguanosine monophosphate (**7MeGPhos**). We used the same reaction conditions used initially and monitored the evolution of the reaction by ^1^H NMR. After the addition of the gold precursor, a grayish precipitate forms within a few minutes. This precipitate is highly insoluble and increases over time. The solid was isolated, but we were unable to further solubilize the compound. We then changed the solvent of the reaction to methanol (instead of DMSO), and also in this case, a highly insoluble precipitate is formed. Further attempts of characterization of the solid were hampered by this lack of solubility; thus, we cannot exclude the formation of the NHC, but we were unable to characterize the solid that is formed upon the reaction of **7MeGPhos** with Au­(I)­SMe_2_Cl (Figure 2).

**Figure 2:**
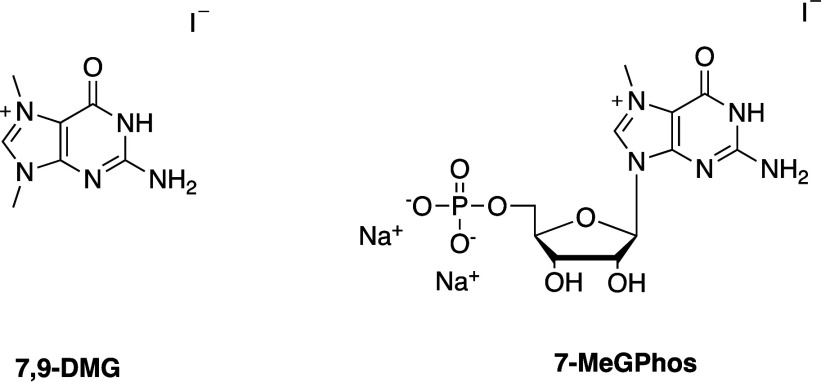
7-Methylguanosinemonophosphate.

The formation of complexes **1**–**4** indicates that the reactivity of guanosine nucleosides to form NHCs, and in particular 7-methylguanosine, is highly dependent on the synthetic method chosen and on the metal precursor employed to stabilize the NHC. Indeed, oxidative addition reactions of guanosine to Pd(0) and Pt(0) require protection of the ribose,^25,26,29^ but deprotonation and formation of gold­(I) compounds do not. We made several attempts to form the corresponding Pd­(II) NHCs using Pd­(OAc)_2_, and also using silver oxide or silver acetate, to no avail, as described earlier. This could be due to a variety of factors, such as the solubility or stability of the final compounds. As it can be observed for the reactivity of **7,9-DMG** with Au­(I)­SMe_2_Cl, variations at the ligand core and the metal employed are pivotal for the outcome of the reaction.

The synthesized gold complexes **1–4** are metalated nucleobases where all the sites involved in Watson–Crick (W:C) base pairing are intact. We examined the base pairing interactions for the guanine–cytosine pair for monocarbene complexes **1** and **3** (Figure 3) using ^1^H NMR, with DMSO-*d*_6_. We chose exclusively the monocarbenes **1** and **3** to enable a direct comparison with similar metalated nucleosides already reported by our group^26^ and to avoid competing sites within the same molecule. Previously, we noted that acetate protection disrupted the formation of the W:C base pair, in particular for the metalated compounds.^26^ We attributed this to the orientation of the sugar within the nucleoside, bringing the acetate groups close to the base pairing sites and preventing hydrogen bonding for the W:C pair. Indeed, for the establishment of the W:C base pair between guanine and cytosine, the difference between the N^1^H downfield shift must be almost twice that observed for the N^2^H_2_ protons, since only one of the hydrogens of N^2^H_2_ is involved in hydrogen bonding.^43,44^ Therefore, Δδ should reflect the average chemical shift of both hydrogens due to the rapid exchange (Figure 3). To ascertain if this effect was also observed, we measured protected monocarbene **1** and unprotected monocarbene **3** and employed similar conditions to those used in previous measurements, i.e., using 20 mM of the complexes in DMSO-*d*_6_ and 1–10 equiv of the complementary nucleoside (Table 1; Table 3 SI).

**Figure 3:**
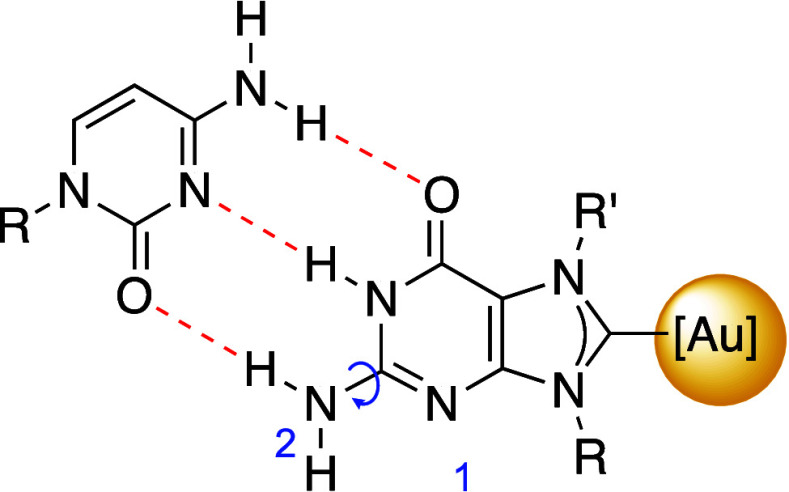
W:C base pair between guanine gold complexes and cytosine.

For complex **1**, with the protected ribose, both the NH and NH_2_ undergo slight downfield shifts upon the addition of cytidine. The Δδ after adding 10 equiv is 0.43 ppm for NH and 0.18 ppm for NH_2_, thus showing a 2:1 ratio evidencing the formation of the W:C base pair. For complex **3**, we proceeded similarly, but we were unable to determine the variation of the NH, since upon addition of the cytidine, the NH signal flattens significantly and we were unable to detect it accurately. For NH_2,_ the Δδ after adding 10 equiv. is 0.23 ppm, similar to the value found for **1**. Importantly, in both cases, we noted the formation of a precipitate upon addition of the cytidine, and when inspecting in the ^1^H NMR the integration values for **1** and **3** versus cytidine, we observed that the amount of the latter present in solution was always higher than the number of equiv effectively added. Given the very low solubility of the gold NHCs, when cytidine is added, some precipitation of the complexes takes place. We cannot therefore compare the values found for gold with those found for the ligand precursors and previously described platinum compounds (Figure 4).

**Figure 4:**
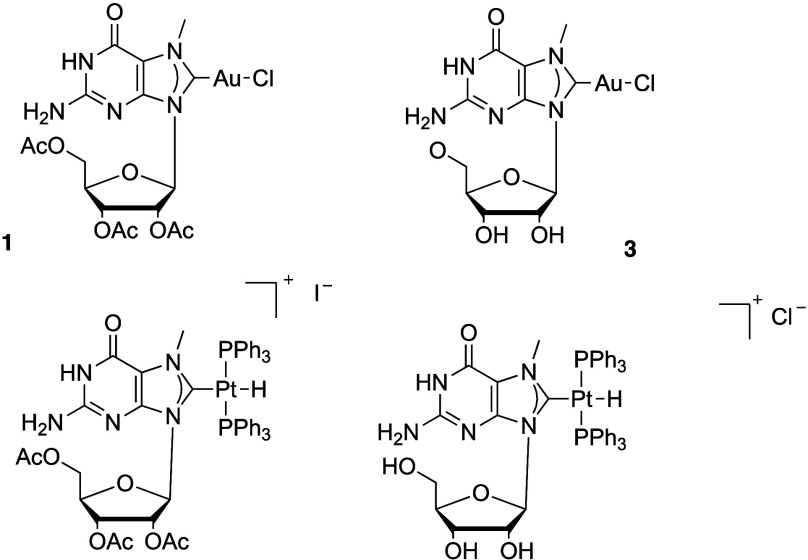
7-Methyl guanosine complexes based on gold and platinum (synthesized previously).

We can nevertheless conclude that for gold mono-NHC complexes **1,** the formation of the W:C base pair is still observed and that the presence of the acetate on the ribose does not disrupt base pairing, contrary to what was observed for analogous platinum compounds bound to the nucleobase via C8.

In summary, gold mono- and bis-NHC complexes based on 7-methylguanosine can be synthesized selectively, starting from the protected or unprotected nucleoside. The gold NHCs complexes are stable in air and in solution for prolonged periods. The gold mono-NHCs are also stable under mildly acidic conditions. Complex **1** is able to retain the ability to form a W:C base pair, in contrast to analogous platinum complexes, for which the acetate protecting group disrupts base pair formation.

## Experimental Procedures

The syntheses of complexes were carried out under an inert atmosphere of N_2_ using Schlenk techniques. All ^1^H and ^13^C­{1H} NMR spectra were recorded at room temperature on Bruker spectrometers (400 MHz). Chemical shifts are reported as δ values in parts per million relative to the deuterated solvent peaks: DMSO-*d*_6_ (δH: 2.50; δC: 39.52). The ligand precursors were synthesized according to the synthetic procedure described previously.^45^ 7-Methylguanosinemonophosphate was synthesized following an adapted procedure. AuSMe_2_Cl was purchased from MCAT GmbH Germany. Mass spectroscopy and elemental analysis were performed by the UniMass Laboratory at Instituto de Tecnologia Qumica e Biológica António Xavier, Portugal.

Complex **1**: A Schlenk tube was charged with methylated and protected guanosine (**7MeG**_**OA**c_, 65 mg, 0.12 mmol), K_2_CO_3_ (16 mg, 0.12 mmol), and disulfide gold chloride (70 mg, 0.24 mmol). After the addition of DMF (3 mL), the reaction mixture was stirred at room temperature for 3 days. The reaction mixture was then filtered through alumina, and the solvent was removed under air flux. The resulting reddish powder was suspended in methanol and filtered. The mother liquors were evaporated, and then acetone was added, affording compound **1** as a white powder (27 mg, 34%). ^1^H NMR (400 MHz, DMSO-*d*_6_): δ 11.80 (bs, 1H, N1-*H*); 7.07 (bs, 2H, N*H*_2_); 6.27 (d, 1H, H-1′, ^3^ *J*_H‑1′,H‑2′_ = 5.1 Hz); 6.14 (dd, 1H, H-2′, ^3^ *J*_H‑2′,H‑1′_ = 5.1 Hz, ^3^ *J*_H‑2′, H‑3′_ = 6.2 Hz); 5.64 (t, 1H, H-3′, ^3^ *J*_H‑3′,H‑2′_ = 6.2 Hz, ^3^J_H‑3′,H‑4′_ = 6.2 Hz); 4.46 (m, 1H, H-5_a_′); 4.37 (m, 1H, H-4′); 4.27 (m, 1H, H-5 _b_′); 3.98 (s, 3H, N7–C*H*_3_); 2.11 (s, 3H, Ac–C*H*_3_); 2.07 (s, 3H, Ac–C*H*_3_); 2.02 (s, 3H, Ac–C*H*_3_) ppm.^13^C­{^1^H} NMR (100 MHz, DMSO-*d*_6_): δ 172.2 (C-8); 170.1 (Ac-*C*O); 169.3 (2C, Ac-*C*O); 156.1 (C-2 or C-6); 154.9 (C-2 or C-6); 150.1 (C-4); 108.3 (C-5); 89.8 (C-1′); 79.6 (C-4′); 71.3 (C-2′); 69.9 (C-3′); 62.7 (C-5′); 37.6 (N7-*C*H_3_); 20.5 (Ac-*C*H_3_); 20.3 (Ac-*C*H_3_); 20.2 (Ac-*C*H_3_) ppm. Anal. calcd for C_34_H_42_O_16_N_10_AuCl: C, 31.14; H, 3.23; N, 10.08; found: C 30.89; H, 3.15; N, 10.60. HRMS-ESI (*m*/*z*): [M+K]^+^ calcd for C_17_H_21_O_8_N_5_AuCl: 694.0375; Obs: 694.0370.

Complex **2**: A Schlenk tube was charged with methylated and protected guanosine (**7MeG** **OA**_c_, 138 mg, 0.25 mmol), K_2_CO_3_ (41 mg, 0.3 mmol), and disulfide gold chloride (35 mg, 0.12 mmol). After the addition of DMF (1.5 mL), the mixture was stirred at room temperature for 3 days. The solvent was removed from the final suspension using air flux. The solid was washed with acetone and dried under vacuum, affording compound **2** as a white powder (67 mg, 48%). ^1^H NMR (400 MHz, DMSO-*d*_6_): δ 12.21 (bs, 1H, N1-*H*); 7.26 (bs, 2H, N*H*_2_); 6.37 (d, 1H, H-1′, ^3^ *J*_H‑1′,H‑2′_ = 4.3 Hz); 5.99 (dd, 1H, H-2′, ^3^ *J*_H‑2′,H‑1′_ = 4.3 Hz, ^3^ *J*_H‑2′,H‑3′_ = 6.4 Hz); 5.70 (dd, 1H, H-3′, ^3^ *J*_H‑3′,H‑2′_ = 6.4 Hz, ^3^ *J*_H‑3′,H‑4′_ = 6.3 Hz); 4.48–4.32 (m, 2H, H-5_a_′+ H-4′); 4.23 (m,1H, H-5_b_′); 4.10 (s, 3H, N7–C*H*_3_); 2.11 (s, 3H, Ac–C*H*_3_); 2.08 (s, 3H, Ac–C*H*_3_); 1.95 (s, 3H, Ac–C*H*_3_) ppm. ^13^C­{^1^H} NMR (100 MHz, DMSO-*d*_6_): δ 184.8 (C-8); 170.1 (Ac-*C*O); 169.6 (Ac-*C*O); 169.4 (Ac-*C*O); 155.5 (2 C, C-2 and C-6); 150.3 (C-4); 108.7 (C-5); 90.1 (C-1′); 79.6 (C-4′); 72.4 (C-2′); 69.9 (C-3′); 63.2 (C-5′); 37.5 (N7-*C*H_3_); 20.4 (Ac-*C*H_3_); 20.3 (2C, Ac-*C*H_3_); ppm. HRMS-ESI (*m*/*z*): [M]^+^ calcd for C_34_H_42_O_16_N_10_Au: 1043.2440; Obs: 1043.2438

Complex **3**: A Schlenk tube was charged with methylated guanosine (**7MeG**, 56 mg, 0.12 mmol), K_2_CO_3_ (16 mg, 0.12 mmol), and disulfide gold chloride (70 mg, 0.24 mmol). After the addition of DMF (3 mL), the reaction mixture was stirred at room temperature for 3 days. The reaction mixture was then filtered through alumina, and the solvent was removed under air flux. The resulting reddish powder was suspended in methanol and filtered. The mother liquor was evaporated, and then acetone was added and the suspension was stirred for a few minutes. The mixture was filtered, and the solid was dried under vacuum, affording compound **3** as a white powder (13 mg, 19%). ^1^H NMR (400 MHz, DMSO-*d*_6_): δ 11.92 (bs, 1H, N1-*H*); 7.01 (bs, 2H, C2–N*H*_2_); 6.07 (m, 1H, H-1′); 5.48 (m, 1H, O*H*); 5.16 (m, 1H, O*H*); 5.07 (m, 2H, H-2′+ O*H*); 4.18 (m, 1H, H-3′); 3.99 (s, 3H, N7–C*H*_3_); 3.90 (m, 1H, H-4′); 3.70 (m, 1H, H-5′); 3.55 (m, 1H, H-5′); ppm. ^13^C­{^1^H} NMR (100 MHz, DMSO-*d*_6_): δ 172.4 (C-8); 155.8 (C-2 or C-6); 154.9 (C-2 or C-6); 150.4 (C-4); 108.3 (C-5); 91.4 (C-1′); 86.1 (C-4′); 71.6 (C-2′); 70.7 (C-3′); 62.2 (C-5′); 37.6 (N7-*C*H_3_) ppm. HRMS-ESI (*m*/*z*): [M+K]^+^ calcd for C_11_H_15_O_5_N_5_AuCl: 568.0058; Obs: 568.0051

Complex **4:** A Schlenk tube was charged with methylated guanosine (**7MeG**, 106 mg, 0.25 mmol), K_2_CO_3_ (41 mg, 0.3 mmol), and disulfide gold chloride (29 mg, 0.1 mmol). After the addition of DMF (1.5 mL), the mixture was stirred at room temperature for 3 days. The solvent was removed from the final suspension using air flux, affording compound **4** as a white powder (49 mg, 45%). ^1^H NMR (400 MHz, DMSO-*d*_6_): δ 6.24 (d, 1H, H-1′, ^3^ *J*_H‑1′,H‑2′_ = 7.2 Hz); 5.22 (bs, 2H, C2–N*H*_2_); 4.97 (t, 1H, H-2′, ^3^ *J*_H‑2′,H‑1′_ = ^3^ *J*_H‑2′,H‑3′_ = 6.17 Hz); 4.17 (m, 1H, H-3′); 4.08 (s, 3H, N7–C*H*_3_); 4.03 (m, 1H, H-4′); 3.70 (m, 1H, H-5′); 3.56 (m, 1H, H-5′); ppm. ^13^C­{^1^H} NMR (100 MHz, DMSO-*d*_6_): δ 182.1 (C-8); 162.8 (C-6); 161.4 (C-2); 149.6 (C-4); 110.3 (C-5); 93.1 (C-1′); 87.1 (C-4′); 71.9 (C-2′); 71.3 (C-3′); 62.4 (C-5′); 36.5 (N7-*C*H_3_) ppm. Anal. calcd for C_22_H_30_O_10_N_10_AuI+H_2_O: C, 27.69; H, 3.59; N, 14.68; found: C 27.61; H, 3.25; N, 14.91. HRMS-ESI (*m*/*z*): [M]^+^ calcd for C_22_H_30_O_10_N_10_Au: 791.1806; Obs: 791.1800.

## Supplementary Material



## Notes

NMR spectra of all compounds; stability tests; and ^1^H NMR studies on formation of Watson–Crick base pairs PDF
