# Supplementary material for: Gold Mono- and Bis-N-heterocyclic Carbenes Based on mRNA cap0
Source: ACS Omega. 2025 Oct 2;10(40):47327–33. doi: 10.1021/acsomega.5c06425 (PMC12529129; doi:10.1021/acsomega.5c06425)
Supplement: Supplementary file 1 [file ao5c06425_si_001.pdf]

Supporting Information

Gold Mono and Bis N-Heterocyclic Carbenes based on mRNA cap0

Giulia Francescato[1], Giulia Orsini[1] and Ana Petronilho*[1]

Instituto de Tecnologia Química e Biológica António Xavier, Universidade Nova de Lisboa.

Av. da República, 2780-157 Oeiras, Portugal

* Correspondence: ana.petronilho@itqb.unl.pt; Tel.: +351-214-469-716

Table of contents

1. NMR spectra 3
1.1. Compound **1** 3
1.2. Compound **2** 4
1.3. Compound **3** 6
1.4. Compound **4** 7
2. Stability tests 8
2.1. Stability of complex **1** in DMSO-d_6 with time and temperature 8
2.2. Stability of complex **1** in DMSO-*d6* in the presence of HCl with time 9
3. ^{1}H NMR studies on formation of Watson-Crick base-pairs 11
3.1. ^{1}H NMR for base-pair interaction between complex **1** and cytidine 12
3.2. ^{1}H NMR for base-pair interaction between complex **3** and cytidine 13

1. NMR spectra

1.1. Compound 1

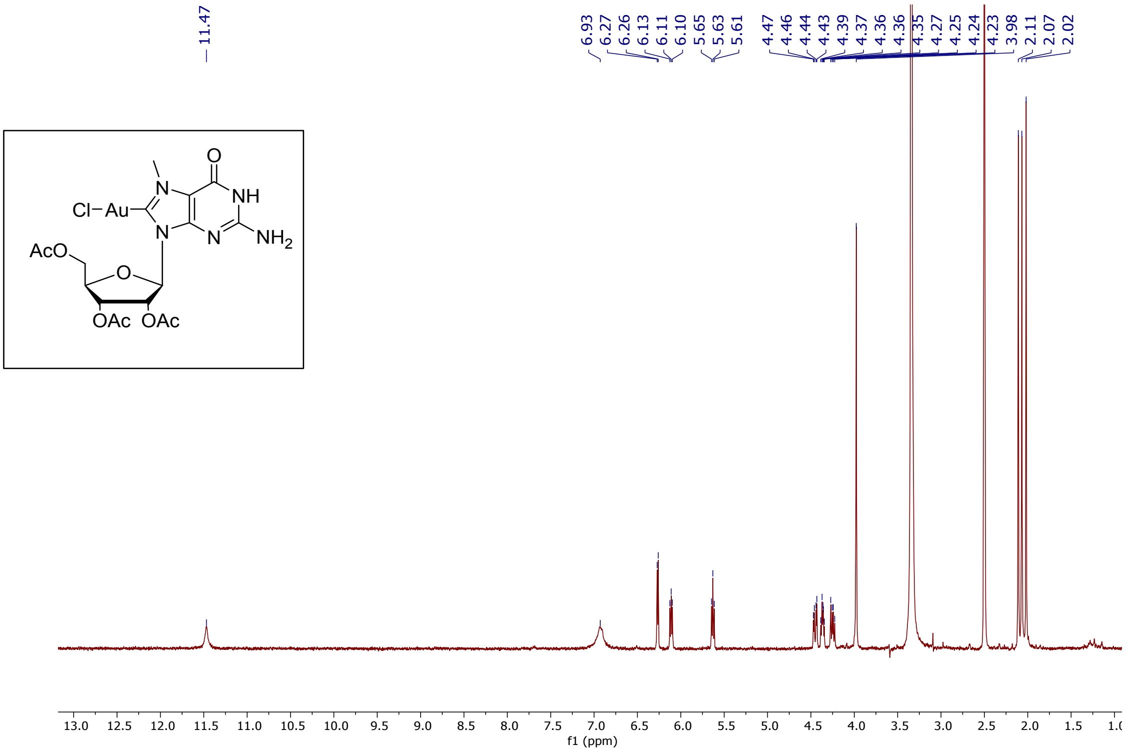

Figure S1. ^{1}H NMR spectrum of compound **1** recorded in DMSO-d_6.

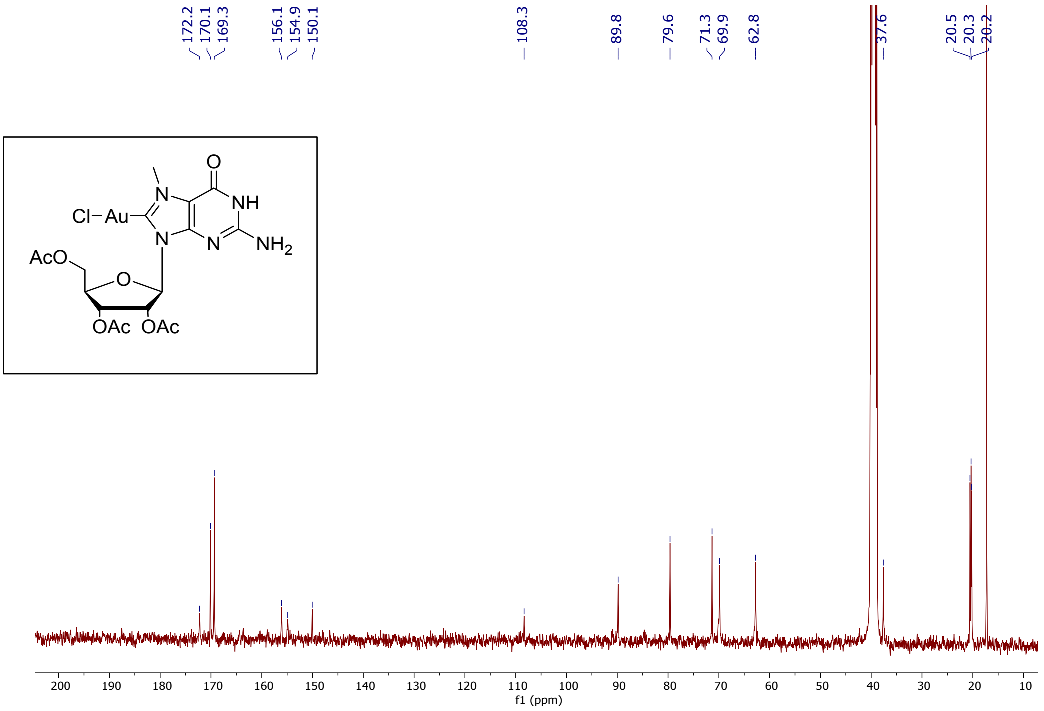

Figure S2. $^{13}C\{^{1}H\}$ NMR spectrum of compound **1** recorded in DMSO-d_6.

1.2. Compound **2**

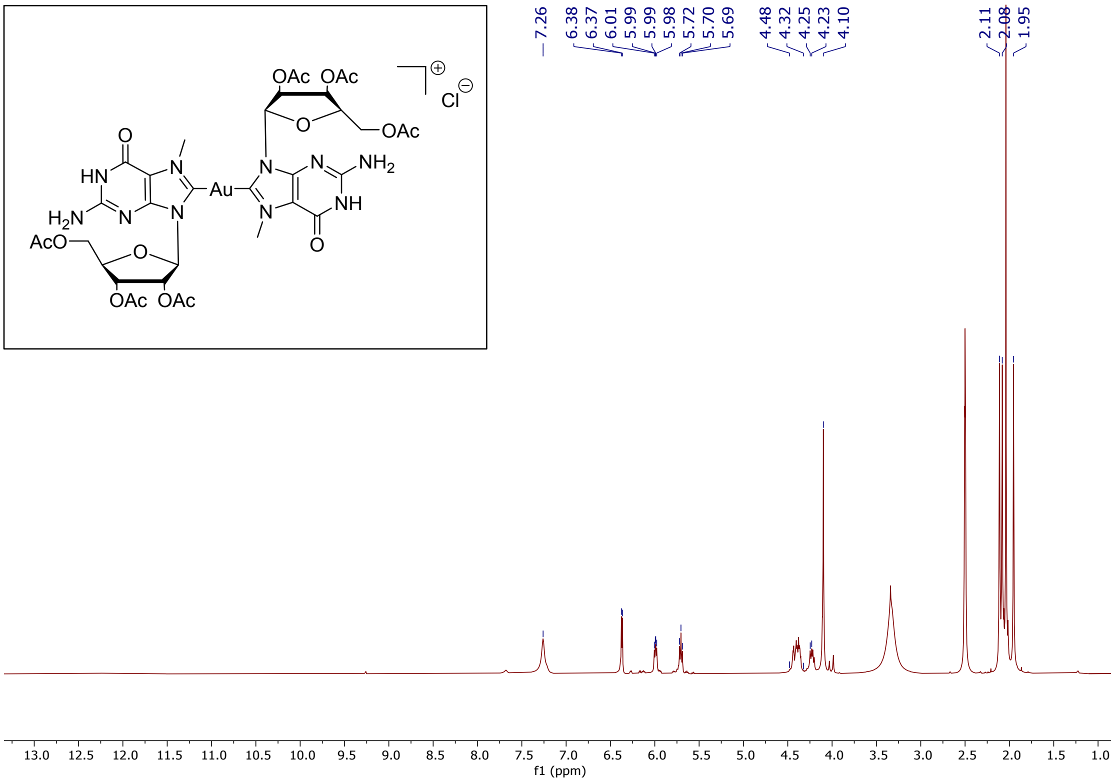

Figure S3. ^{1}H NMR spectrum of compound **2** recorded in DMSO-d_6.

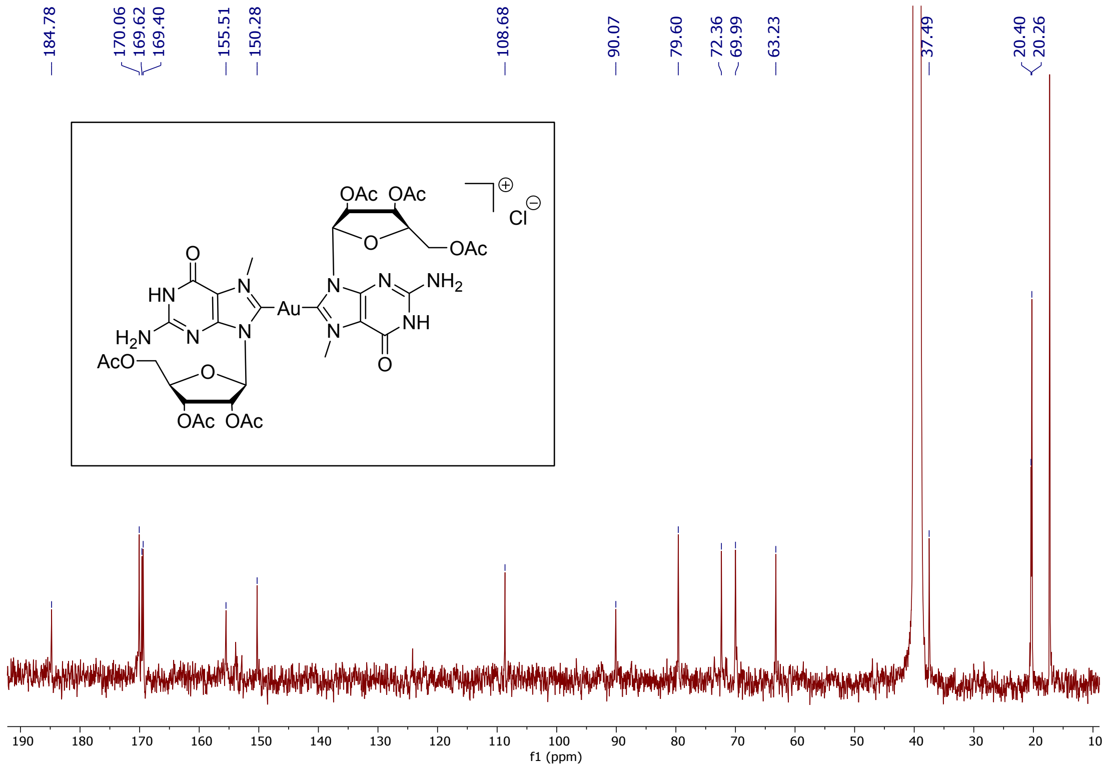

Figure S4. ^{13}C{^{1}H} NMR spectrum of compound **2** recorded in DMSO-d_6.

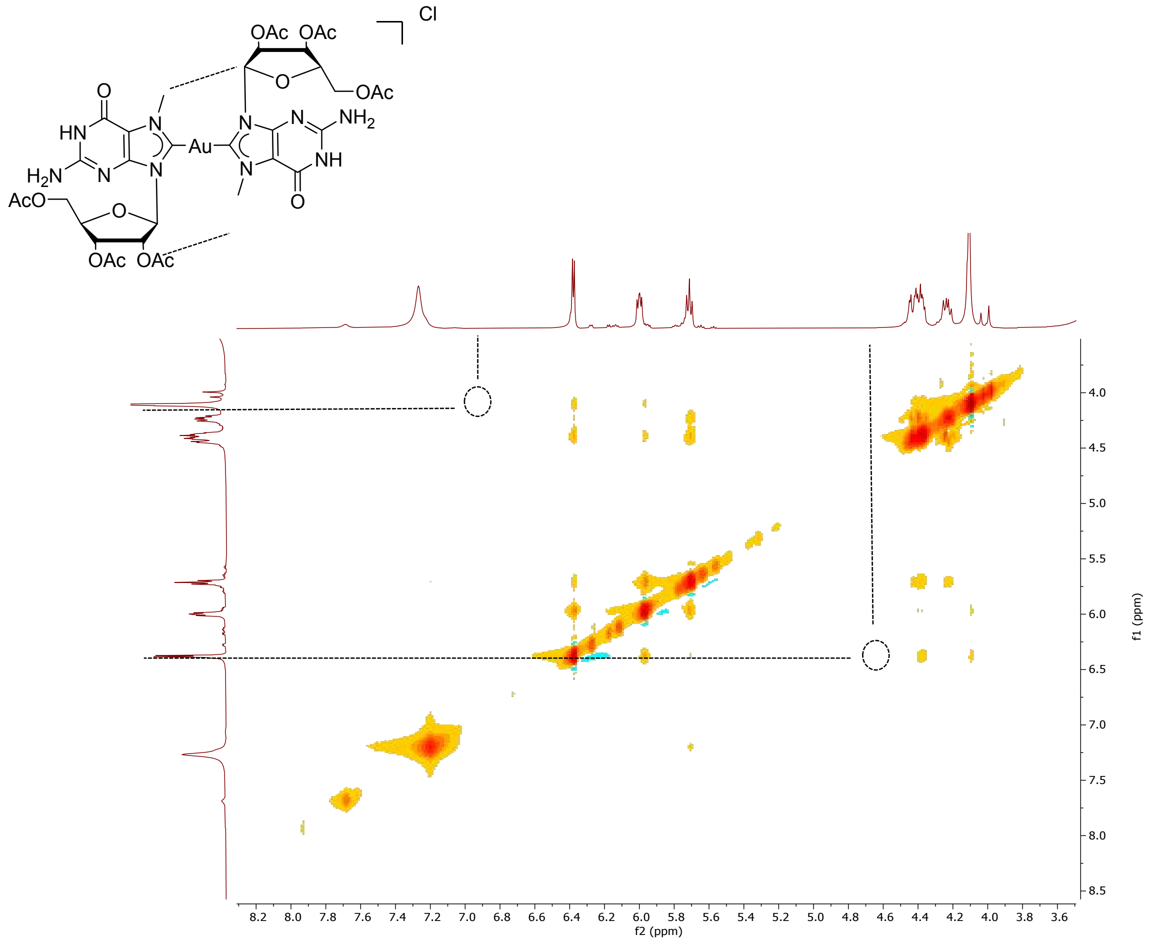

Figure S5. NOESY spectrum of compound **2** recorded in DMSO-d_6.

1.3. Compound **3**

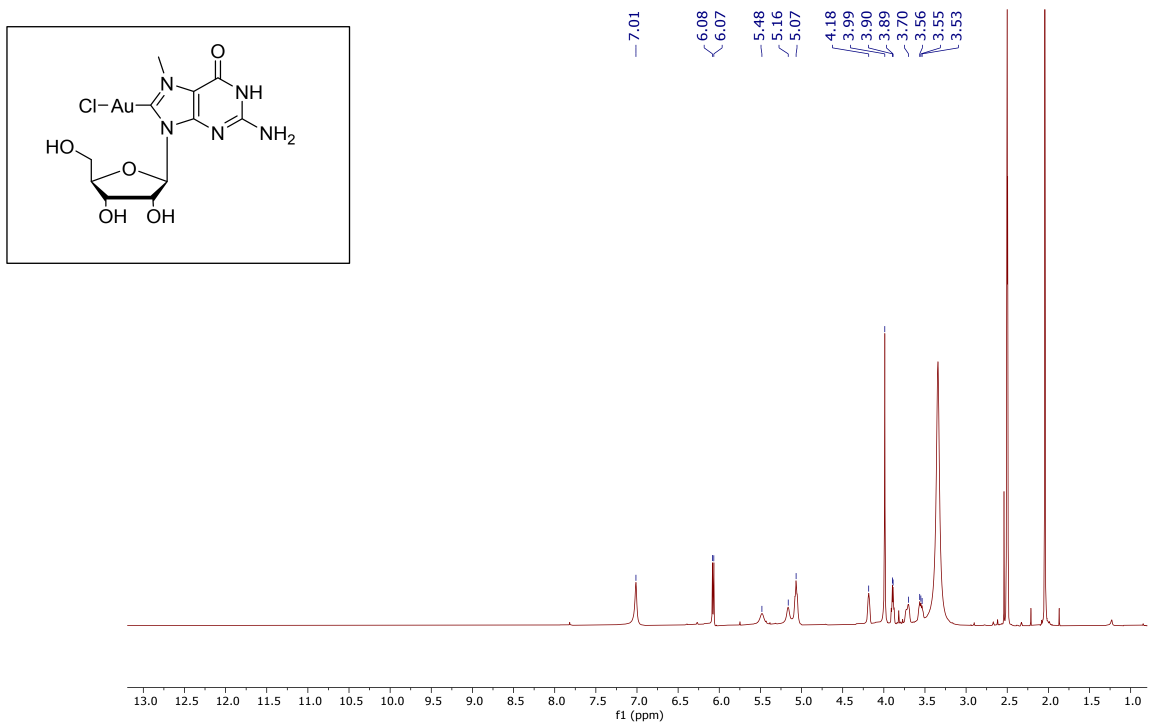

Figure S6. ^{1}H NMR spectrum of compound **3** recorded in DMSO-d_6

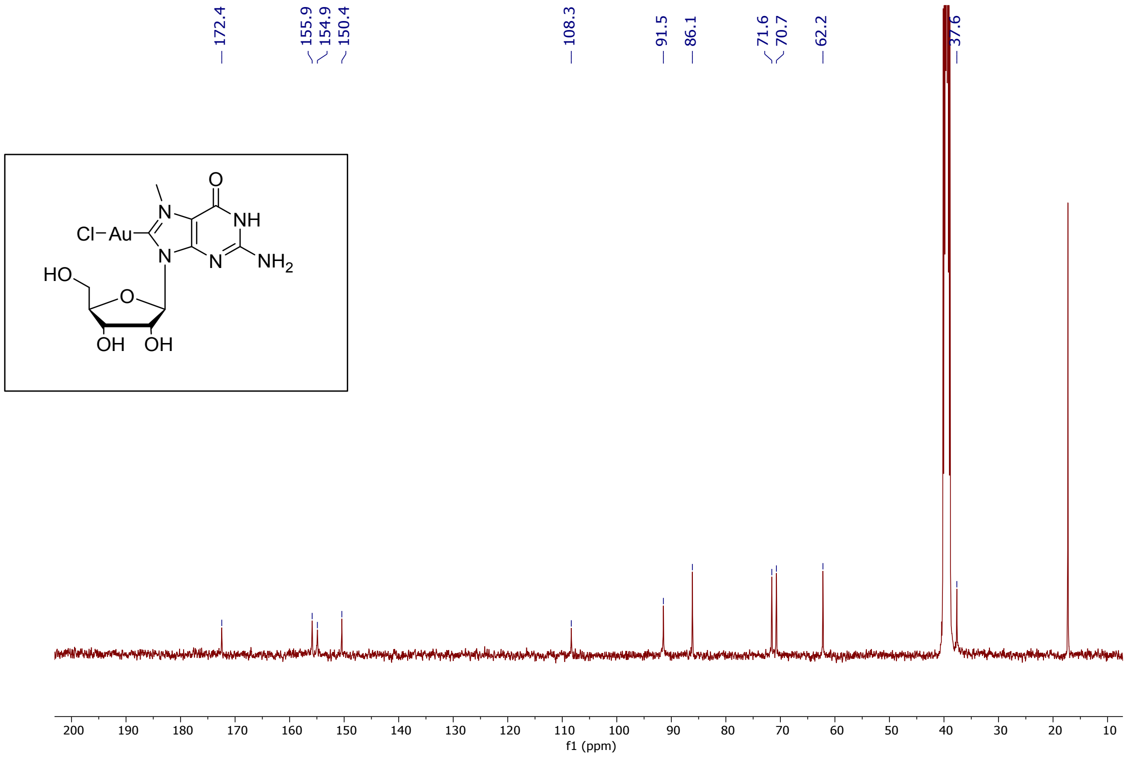

Figure S7. $^{13}C\{^{1}H\}$ NMR spectrum of compound **3** recorded in DMSO-d_6.

1.4. Compound **4**

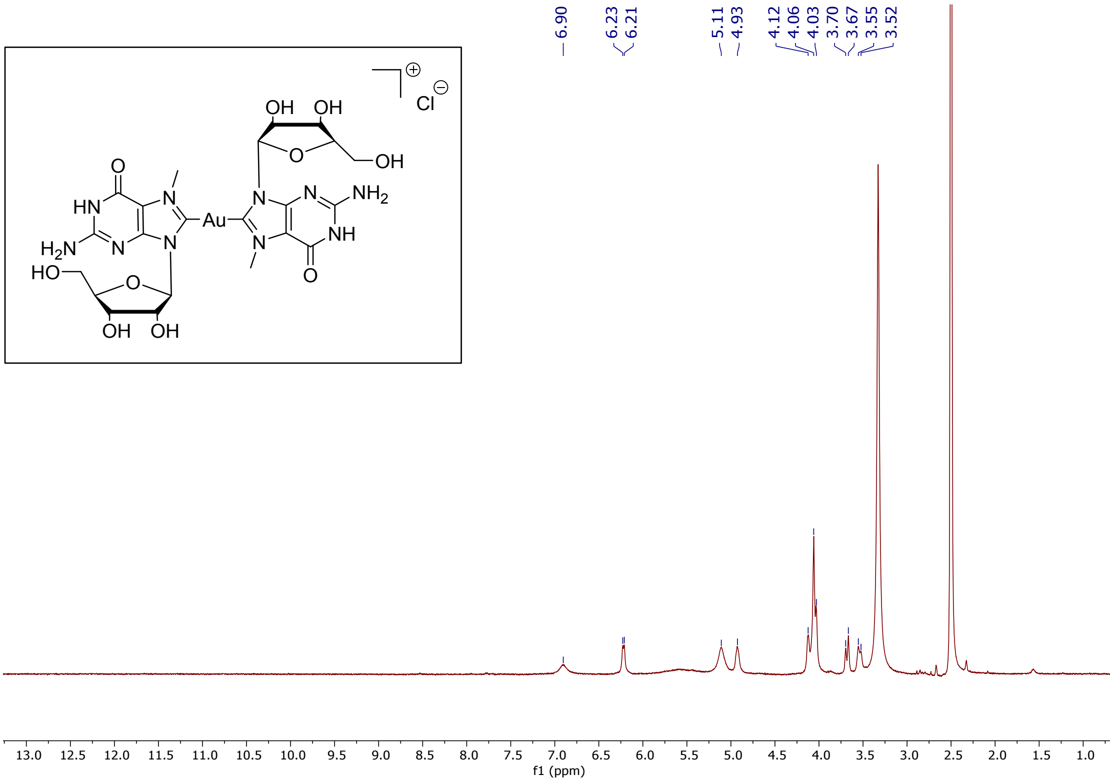

Figure S8. ^{1}H NMR spectrum of compound **4** recorded in DMSO-d_6.

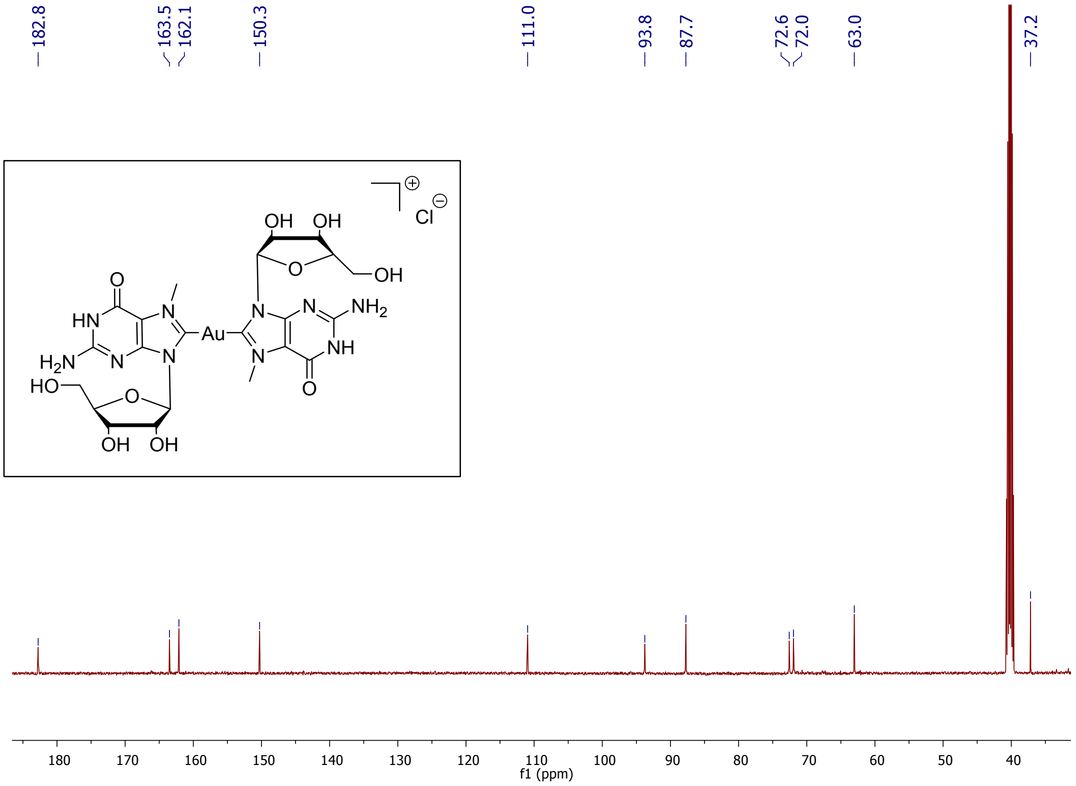

Figure S9. $^{13}C\{^{1}H\}$ NMR spectrum of compound **4** recorded in DMSO-d_6.

2. Stability tests

2.1. Stability of complex 1 in DMSO-d_6 with time and temperature

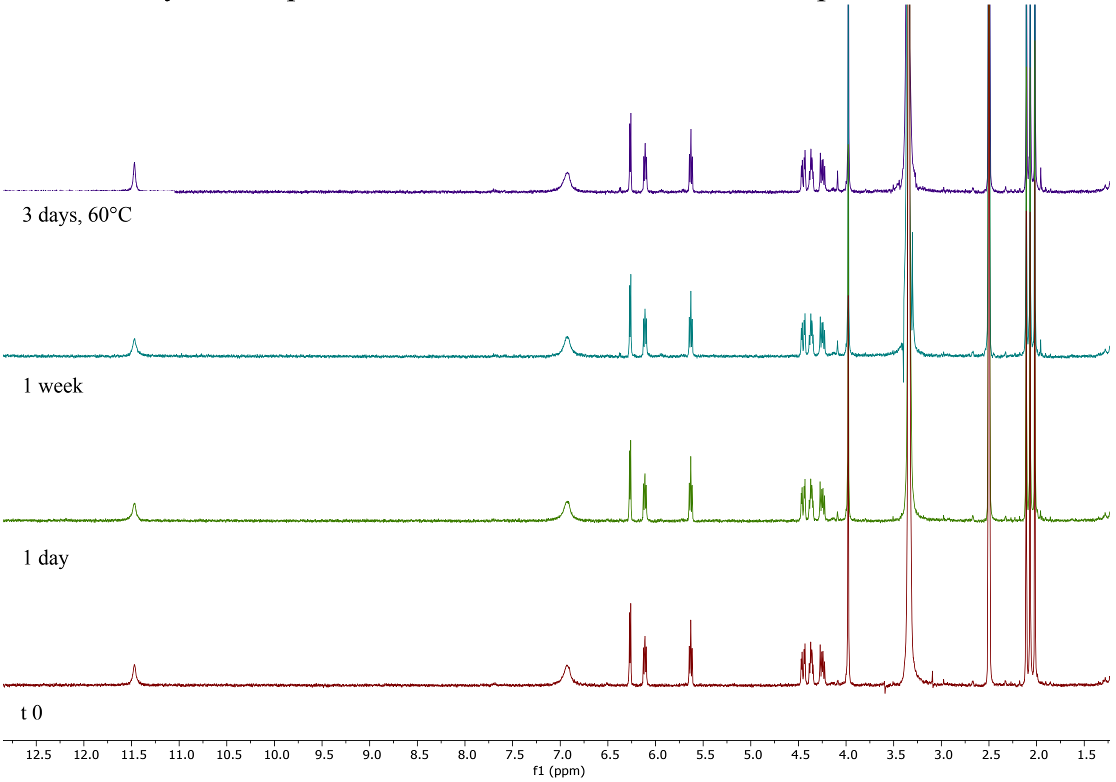

Figure S10. ^{1}H NMR spectrum of compound **1** in DMSO-d_6 at different times and different temperatures.

2.2. Stability of complex **1** in DMSO-*d6* in the presence of HCl with time

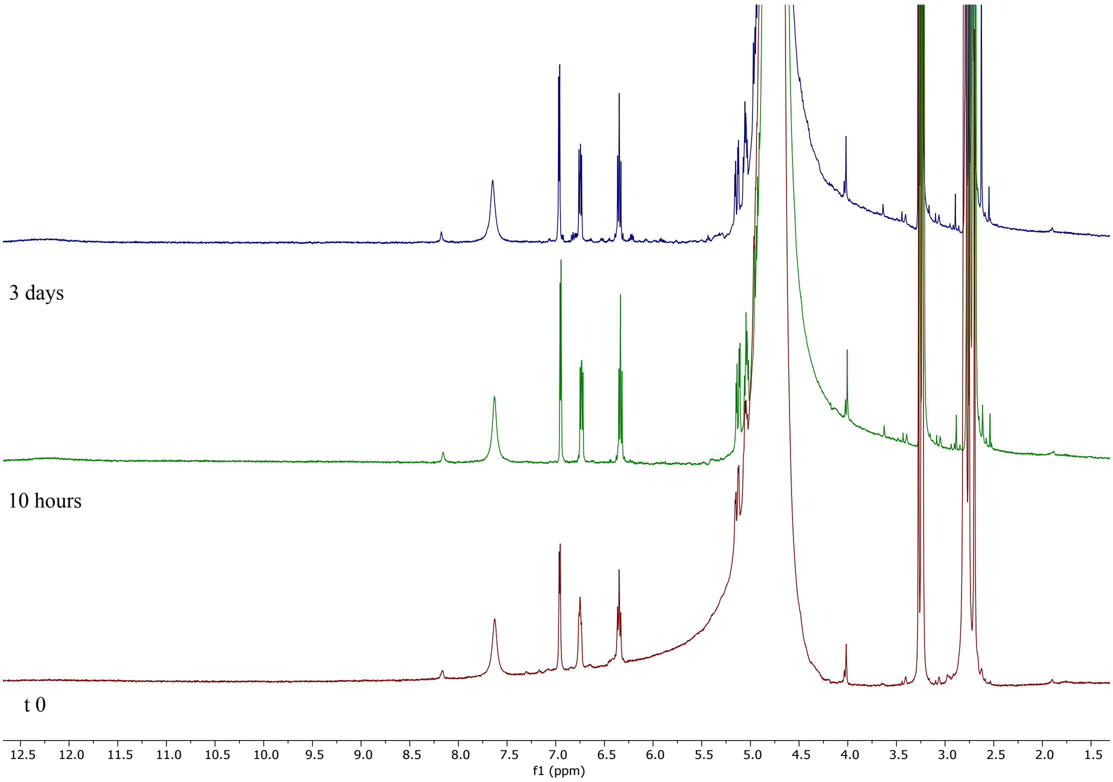

Figure S10. ^{1}H NMR spectrum of compound **1** in DMSO *d6* at different times in the presence of 100 µL of an aqueous HCl solution (pH 2).

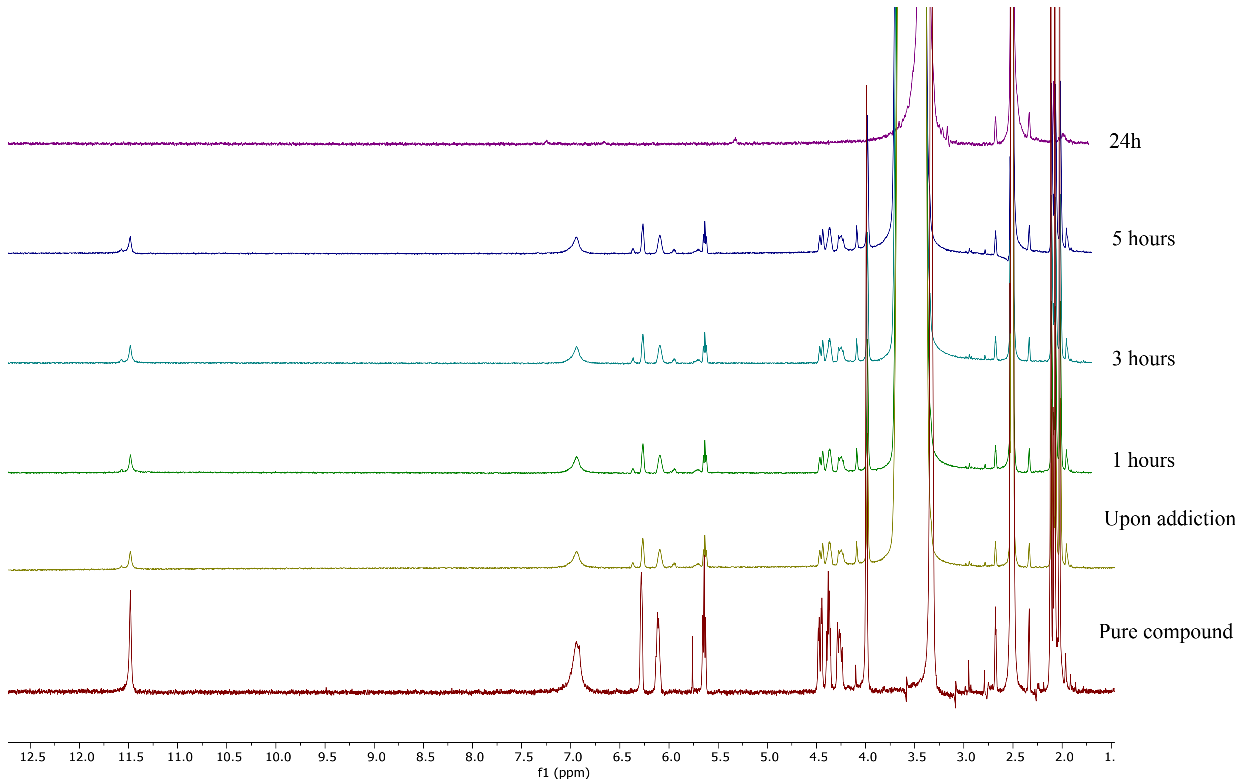

Figure S11. ^{1}H NMR spectrum of compound **1** in DMSO *d6* at different times in the presence of 100 μL of an aqueous HCl solution (pH 1).

3. ^{1}H NMR studies on formation of Watson-Crick base-pairs

Table S1. Variation of the chemical shits measured using ^{1}H NMR in DMSO-*d*6 for complexes **1** and **3** in the presence of increasing amounts of cytidine (**Cy**).

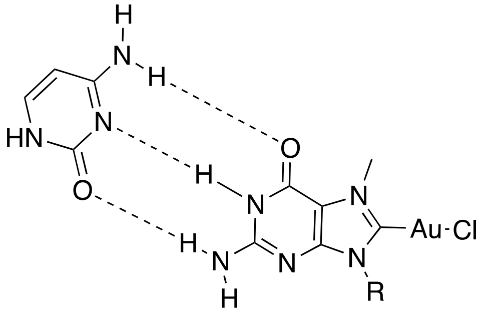

Compound	Group	**Cy** (equiv.) 0	1	3	5	8	10	Δδmax / ppm	
**1**	1-NH	11,77	11,85	11,93	12,02	12,17	12,20	0,43	
	NH_2	7,16	7,19	7,22	7,27	7,31	7,34	0,18	
**3**	1-NH	11.49		-	-	-	-	-	-
	NH_2	6,74.	6,82	6,88	6,90	6,91	6,97	0,23	

3.1. ^{1}H NMR for base-pair interaction between complex **1** and cytidine

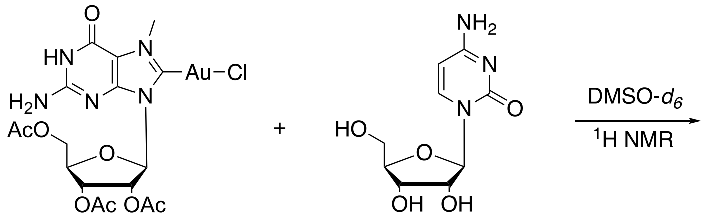

Scheme S1. Watson-Crick base-pairs between complex **1** and Cytidine

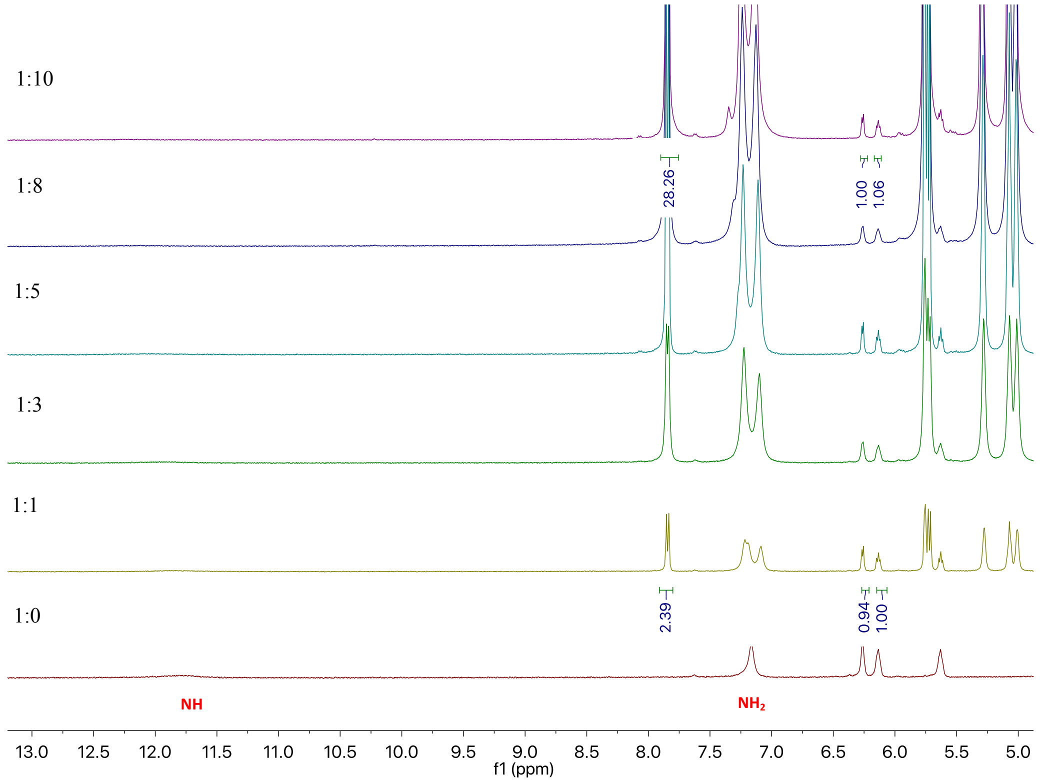

Figure S13. ^{1}H NMR spectrum of complex **1**, in deuterated DMSO at room temperature and in presence of different concentrations of **Cy** (0, 1, 3, 5, 8 and 10 equivalents in respect with the amount of the guanosine derivative **1**). $C_{complex}$ **1** = 0.02 M

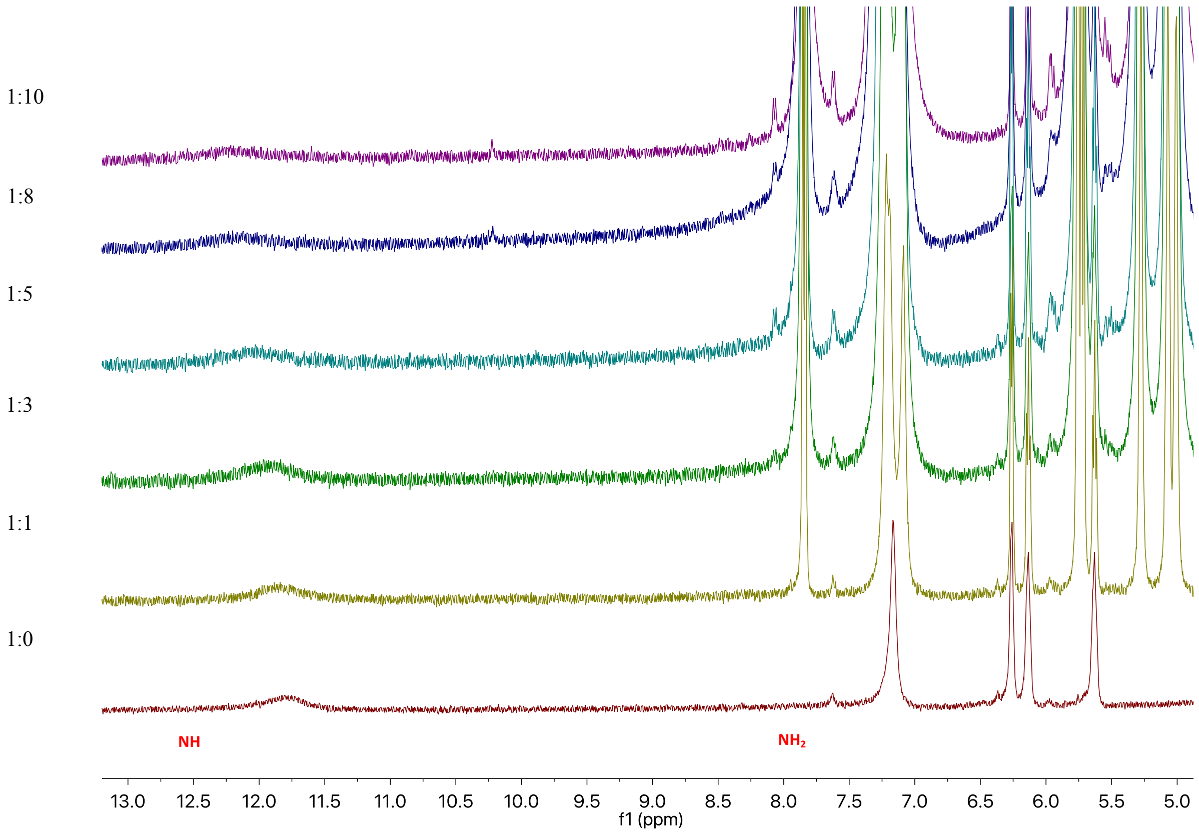

Figure S14 ^{1}H NMR spectrum of selected areas covering the NH and NH_2 groups (15 to 6.5 ppm) of complex **1**, in deuterated DMSO at room temperature and in presence of different concentrations of **Cy** (0, 1, 3, 5, 8 and 10 equivalents in respect with the amount of the guanosine derivative **1**). $C_{complex}$ **1** = 0.02 M.

3.2. ^{1}H NMR for base-pair interaction between complex **3** and cytidine

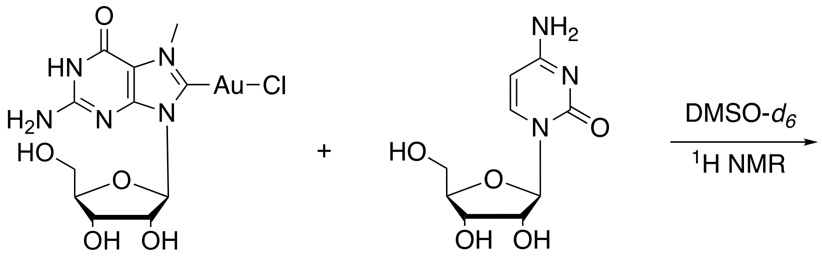

Scheme S2. Watson-Crick base-pairs between complex **3** and Cytidine

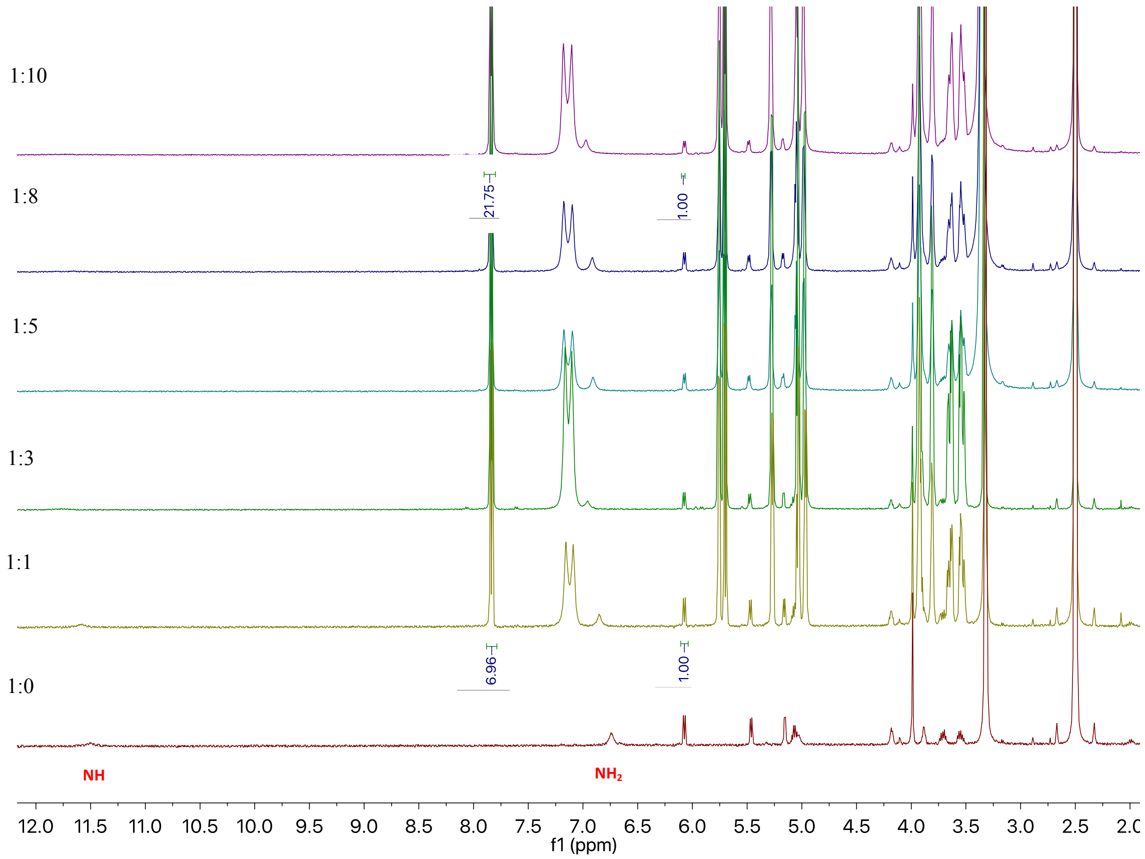

Figure S15. ^{1}H NMR spectrum of complex **3**, in deuterated DMSO at room temperature and in presence of different concentrations of **Cy** (0, 1, 3, 5, 8 and 10 equivalents in respect with the amount of the guanosine derivative **3**). $C_{complex}$ **3** = 0.02M

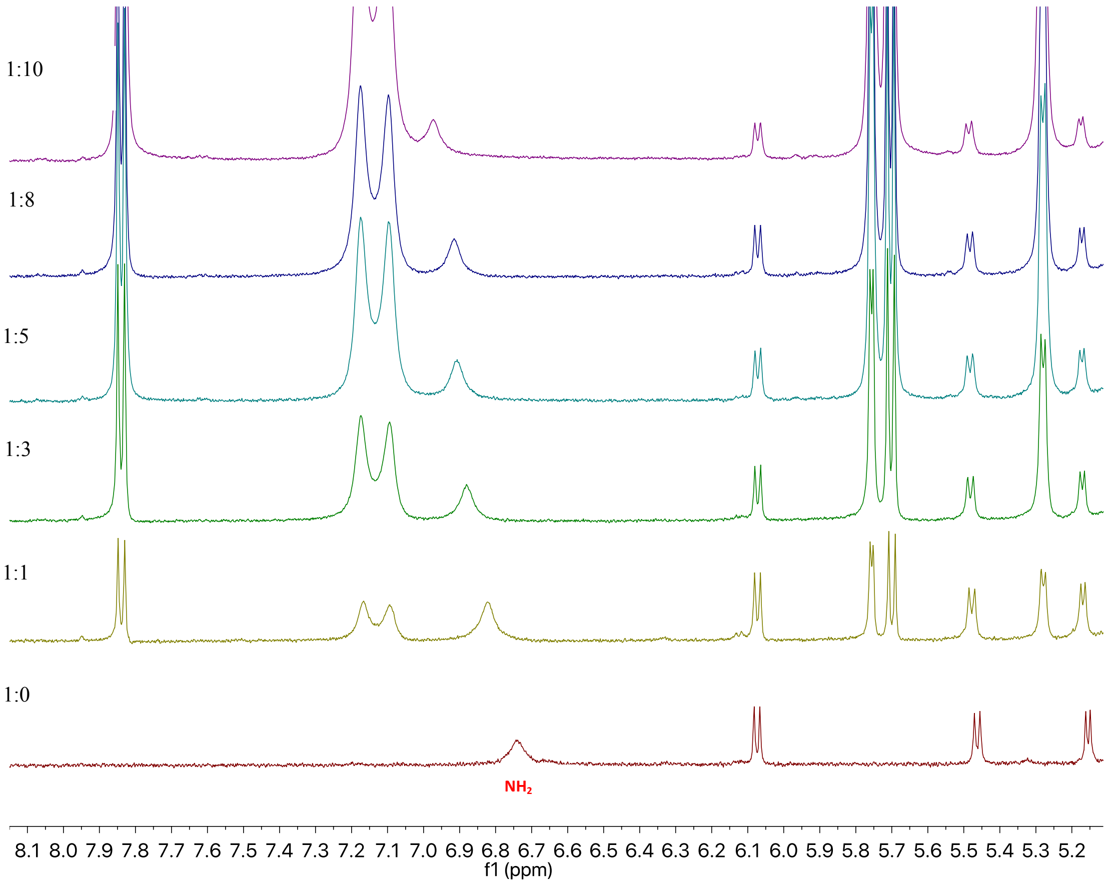

Figure S16. ^{1}H NMR spectrum of selected areas covering the NH_2 groups (8.5 to 4.5 ppm) of complex **3**, in deuterated DMSO at room temperature and in presence of different concentrations of **Cy** (0, 1, 3, 5, 8 and 10 equivalents in respect with the amount of the guanosine derivative **3**). $C_{complex}$ **3** = 0.02M.
